# Road traffic crashes, injury and fatality trends in Sri Lanka: 1938–2013

**DOI:** 10.2471/BLT.14.150193

**Published:** 2015-06-25

**Authors:** Samath D Dharmaratne, Achala Upendra Jayatilleke, Achini C Jayatilleke

**Affiliations:** a Department of Community Medicine, University of Peradeniya, Peradeniya, Sri Lanka.; b Postgraduate Institute of Medicine, University of Colombo, 160 Prof. Nandadasa Kodagoda Mawatha, Colombo 7, 00700, Sri Lanka.; c The Family Planning Association of Sri Lanka, Colombo, Sri Lanka.

## Abstract

**Objective:**

To analyse trends in road traffic crashes, injuries and fatalities over 75 years in Sri Lanka.

**Methods:**

Data on road traffic crashes, injuries and fatalities between 1938 and 2013 were obtained from the Police Statistics Unit. Rates per 100 000 population were calculated and trends were analysed using joinpoint regression analysis.

**Findings:**

Road traffic crashes and injuries rose substantially between 1938 and 2013: annual crashes increased from 61.2 to 183.6 per 100 000 people; injuries, from 35.1 to 98.6 per 100 000; and fatalities, from 3.0 to 10.8 per 100 000 people per year. Joinpoint analysis showed large fluctuations in crashes and injuries over time but the fatalities rose almost continuously. These fluctuations paralleled the country’s political and economic development. In some years, better traffic law enforcement and improved public transportation may have been associated with reduced crashes and injuries, whereas rapid growth in vehicle numbers, especially two- and three-wheeled vehicles, may have contributed to increased crashes and injuries. In addition, insurance policies that did not require a police report to claim may have led to underreporting of crashes and allowed drivers to avoid prosecution.

**Conclusion:**

Fluctuations over time in road traffic crashes and injuries in Sri Lanka are associated with changes in political, economic and traffic policy. There is potential for reducing road traffic crashes and injuries through better traffic law enforcement, restrictions on the importation of two- and three-wheeled vehicles and policies to improve road safety and prevent underreporting of crashes.

## Résumé

**Objectif:**

Analyser les tendances en matière d'accidents, de blessures et de mortalité de la route au Sri Lanka sur 75 ans.

**Méthodes:**

Les données sur les accidents, les blessures et la mortalité de la route entre 1938 et 2013 proviennent de l'unité des statistiques de la police. Nous avons calculé leur taux pour 100 000 personnes et analysé les tendances à l'aide d'une analyse de régression par points de jonction.

**Résultats:**

Les accidents et les blessures de la route ont considérablement augmenté entre 1938 et 2013: le nombre d’accidents est passé de 61,2 à 183,6 pour 100 000 personnes, celui des blessures de 35,1 à 98,6 pour 100 000 personnes et la mortalité de 3,0 à 10,8 pour 100 000 personnes par an. L'analyse par points de jonction a montré d'importantes fluctuations du nombre d’accidents et de blessures au fil du temps mais la mortalité a augmenté pratiquement de manière continue. Ces fluctuations vont de pair avec le développement politique et économique du pays. En quelques années, la meilleure application des règles de circulation routière ainsi que l'amélioration des transports publics peuvent avoir eu un lien avec la réduction des accidents et des blessures, tandis que la croissance rapide du nombre de véhicules, en particulier à deux et trois roues, peut avoir contribué à l'augmentation des accidents et des blessures. En outre, les polices d'assurance qui n'exigent pas la présentation d'un rapport de police pour déclarer un sinistre peuvent avoir entraîné un sous-signalement des accidents et avoir permis aux conducteurs d'éviter des poursuites.

**Conclusion:**

Les fluctuations du nombre d’accidents et de blessures de la route au fil du temps au Sri Lanka sont associées aux changements d'orientations politiques, économiques et en matière de circulation. Il est possible de réduire les accidents et les blessures de la route grâce à une meilleure application des règles de circulation, à des restrictions sur l'importation de véhicules à deux et trois roues ainsi qu'à des politiques visant à améliorer la sécurité routière et à éviter le sous-signalement des accidents.

## Resumen

**Objetivo:**

Analizar las tendencias en las colisiones, las lesiones y las muertes en las vías de tránsito en Sri Lanka durante el transcurso de 75 años.

**Métodos:**

Se obtuvieron datos sobre las colisiones, las lesiones y las muertes en las vías de tránsito entre 1938 y 2013 a través la unidad de estadísticas de la policía. Se calcularon las tasas por 100.000 habitantes y se analizaron las tendencias mediante la utilización de análisis de regresión de puntos de inflexión.

**Resultados:**

El número de colisiones y lesiones en las vías de tránsito creció substancialmente entre 1938 y 2013: las colisiones anuales crecieron de un 61,2 a un 183,6 por 100.000 personas; las lesiones, de un 35,1 a un 98,6 por 100.000 personas; y las muertes de un 3,0 a un 10,8 por 100.000 personas al año. Los análisis de puntos de inflexión mostraron grandes fluctuaciones en las colisiones y las lesiones a lo largo del tiempo, pero las muertes crecieron prácticamente de forma continua. Estas fluctuaciones eran paralelas al desarrollo político y económico del país. En algunos años, puede asociarse la reducción de colisiones y lesiones a una mejor aplicación de las leyes de tráfico y una mejora en el transporte público, mientras que el rápido crecimiento en el número de vehículos, especialmente aquellos de dos y tres ruedas, puede haber contribuido en el aumento de las colisiones y las lesiones. Además, las pólizas de seguros que no requieren la solicitud de un informe policial pueden haber llevado a una deficiente notificación de las colisiones y permitido que los conductores evitaran la acusación.

**Conclusión:**

Las fluctuaciones con el tiempo en las colisiones y las lesiones en las vías de tránsito en Sri Lanka están asociadas a los cambios en las políticas económicas, políticas y viales. Existe potencial para reducir las colisiones y las lesiones en vías de tránsito a través de una mejor aplicación de las leyes de tráfico, restricciones en la importación de vehículos de dos y tres ruedas y una mejora de la seguridad vial para evitar la deficiente notificación de los accidentes.

## ملخص

**الغرض:**

تحليل النزعات في الحوادث والإصابات والوفيات المرورية على الطرق في غضون 75 عامًا في سريلانكا.

**الطريقة:**

تم الحصول على المعطيات حول الحوادث والإصابات والوفيات المرورية على الطرق في الفترة ما بين عامي 1938 و2013 من وحدة الإحصاءات في الشرطة. وتم قياس معدلات كل شريحة سكانية تضم 100,000 شخص، كما تم تحليل النزعات بواسطة تحليل التحوف (Regression Analysis) من برنامج Joinpoint.

**النتائج:**

لقد ارتفعت نسبة حوادث التصادم المروري والإصابات الناتجة عنها على الطرق بشكل كبير في الفترة ما بين عامي 1938 و2013: فقد زادت الحوادث السنوية للتصادم المروري من 61.2 إلى 183.6 لكل مجموعة تضم 100,000 شخص؛ وزادت الإصابات من 35.1 إلى 98.6 لكل مجموعة تضم 100,000 شخص؛ وزادت الوفيات من 3.0 إلى 10.8 لكل مجموعة تضم 100,000 شخص. وأظهر تحليل Joinpoint تقلبات كبيرة في حوادث التصادم والإصابات بمرور الوقت إلا أن معدل الوفيات شهد ارتفاعًا بشكل شبه مستمر. وكانت هذه التقلبات متوازية مع التنمية السياسية والاقتصادية في البلاد. ومن المحتمل أن تكون زيادة الصرامة في إنفاذ لقانون المرور وتطوير وسائل النقل العام قد أديا في بعض السنوات إلى انخفاض الحوادث والإصابات، في حين أن هناك احتمالاً لأن يكون النمو السريع في أعداد المركبات – خاصة ما يسير منها على عجلتين أو ثلاث عجلات – قد أسهم في زيادة الحوادث والإصابات. بالإضافة إلى ذلك، ربما تكون وثائق التأمين التي لم تكن تتطلب تقديم تقرير من الشرطة للمطالبة بالتعويض قد أدت إلى النقص في الإبلاغ عن الحوادث والسماح للسائقين بتجنب الملاحقة القضائية.

**الاستنتاج:**

لقد ارتبطت التقلبات التي تطرأ بمرور الوقت على الحوادث والإصابات المرورية على الطرق في سريلانكا بالتغيرات في الاتجاهات السياسية والاقتصادية والسياسات المرورية. هناك إمكانية للحد من حوادث التصادم والإصابات المرورية على الطرق من خلال إنفاذ قانون المرور بشكل أكثر صرامة، وفرض قيود على استيراد المركبات التي تسير على عجلتين أو ثلاث عجلات، وإقرار السياسات الداعية إلى تحسين السلامة على الطرق ومنع القصور في الإبلاغ عن الحوادث.

## 摘要

**目的:**

旨在分析斯里兰卡 75 年以来的道路交通事故、受伤和死亡趋势。

**方法:**

从警方统计部门处获得从 1938 年至 2013 年之间的道路交通事故、受伤和死亡数据。 我们计算了每 10 万人口的比率，并采用连接点回归分析法分析趋势。

**结果:**

道路交通事故和受伤率在 1938 年至 2013 年期间大幅増长： 每 10 万人口的年度事故率从 61.2 增长至 183；每 10 万人口的受伤率从 35.1 增长至 98.6；每 10 万人口的年度死亡率从 3.0 增长至 10.8。 连接点分析显示一段时间内的事故率和受伤率会出现大幅波动，但死亡率几乎呈现持续上升的趋势。 这些波动情况与国家的政治和经济发展相一致。 在一些年份中，交通执法得以优化且公共交通得到改善这两点会相应地降低事故率和受伤率，但是，汽车（尤其是两轮车和三轮车）数量的迅速增加导致事故和受伤情况加剧。 此外，因保险政策无需出具警方索赔报告，这样便会导致少报事故，也会让司机免遭起诉。

**结论:**

斯里兰卡境内的道路交通事故和受伤率在一段时间内的浮动与政治、经济和交通政策的改变有关。 我们可以优化交通执法，限制两轮车和三轮车的引进，同时制定政策来提高道路安全并防止少报事故，从而降低事故率和受伤率。

## Резюме

**Цель:**

Проанализировать тенденции показателей дорожно-транспортных происшествий, травматизма и смертности в Шри-Ланке за 75 лет.

**Методы:**

Данные о дорожно-транспортных происшествиях, травматизме и гибели людей в период между 1938 и 2013 г. были получены в отделе статистики полиции. В ходе исследования были рассчитаны показатели на 100 000 населения и рассмотрены тенденции с использованием регрессионного анализа по точкам перегиба.

**Результаты:**

Количество дорожно-транспортных происшествий и травм в них значительно выросло в период с 1938 по 2013 г. Ежегодный показатель дорожно-транспортных происшествий вырос с 61,2 до 183,6 случая на 100 000 человек, показатель травматизма увеличился с 35,1 до 98,6 на 100 000 человек, а показатель смертности — с 3,0 до 10,8 случая на 100 000 человек в год. Анализ по точкам перегиба показал наличие значительных колебаний в показателях происшествий и травм в них с течением времени, но показатель смертности рос практически непрерывно. Колебания показателей соответствовали изменениям в политическом и экономическом развитии страны. В некоторые годы ужесточение контроля за соблюдением правил дорожного движения и улучшение ситуации с общественным транспортом могли ассоциироваться с уменьшением количества происшествий и травм. Однако быстрый рост количества транспортных средств, в особенности двух- и трехколесных, внес свой вклад в рост показателей дорожно-транспортных происшествий и травматизма в них. Кроме того, договоры страхования, которые не требуют полицейского отчета для подачи иска, могли привести к тому, что количество дорожно-транспортных происшествий занижалось, что помогало водителям избегать преследования.

**Вывод:**

С течением времени колебания показателей частоты дорожно-транспортных происшествий и травматизма в Шри-Ланке связываются с изменениями в политике, экономике страны и правилах дорожного движения. Существует возможность уменьшения числа дорожно-транспортных происшествий и травматизма в них за счет ужесточения контроля за исполнением правил дорожного движения, ограничений на импорт двух- и трехколесных транспортных средств и ужесточения правил страхования. Комплекс перечисленных мер будет способствовать безопасности на дорогах и предотвращению сокрытия фактов дорожно-транспортных происшествий.

## Introduction

Road traffic injuries are a major but neglected global public health problem.^1^ Each year, road traffic crashes are responsible for over 1 million deaths and 20 to 50 million injuries worldwide.^1^^,^^2^ Low- and middle-income countries are the most affected, because road traffic crashes and injuries are linked not only to the number of vehicles, road conditions and drivers’ behaviour but also to the country’s level of economic and social development.^1^^–^^3^ In particular, poor road infrastructure, inappropriate mixing of vehicle types, inadequate traffic law enforcement and delayed implementation of road safety policies can increase road traffic crashes.^3^

Sri Lanka is a lower-middle-income country in south Asia that has a substantial burden of road traffic injuries and fatalities.^4^^–^^7^ Between 1938 and 1997, the absolute number of road traffic fatalities in the country increased 10-fold to reach 1835 deaths in 1997 in a population of around 18 million.^5^^,^^7^ Despite the need for immediate action to reduce this growing burden, there is a paucity of coordinated government road safety strategies and road safety research in the country.^7^

Sri Lanka has an interesting traffic history. Between 1815 and 1948, the island was governed by the United Kingdom of Great Britain and Northern Ireland. The British expanded the road infrastructure,^8^ increased the number of vehicles and developed railways to transport goods.^9^ In 1948, Sri Lanka gained its independence and the new government further improved the road infrastructure. However, because railways were neglected, road transport was used for goods, increasing the risk of crashes. Sri Lanka passed its first traffic act in 1934 and, from 1938 onwards, the police documented traffic accidents.^10^^,^^11^ In 1951, the country mandated that all motor vehicles be registered with the Department of Motor Traffic,^8^^,^^12^ which meant that vehicles had to be roadworthy. The law specifically prohibited the registration of unlawfully fabricated motor vehicles, which were common at the time. In 1953, Sri Lanka established a separate traffic police division.^10^^,^^11^ This increased the number of traffic police and improved traffic law enforcement.

In 1977, Sri Lanka introduced an open economic policy that promoted motor vehicle imports. This resulted in a massive influx of motorcycles and three-wheeled taxis,^8^ which are prone to crashes because they are unstable and topple easily. Since two- and three-wheeled vehicles are less robust than other vehicles, occupants are more likely to be injured in a crash.^1^^–^^3^ Finally, despite the large increase in motor vehicles, the road infrastructure did not develop at the same pace.

In Sri Lanka, a lack of road safety research and the limited availability of statistics on road traffic crashes and injuries made it difficult for policy-makers to propose interventions that would prevent road traffic crashes. The aims of this study were to describe the trends in road traffic crashes, injuries and fatalities in Sri Lanka from 1938 to 2013 and to identify factors associated with these trends. 

## Methods

Our analysis used statistics on road traffic crashes and road traffic injuries from the road traffic crash statistics’ database maintained by the traffic police headquarters, which is the only comprehensive such database in Sri Lanka.^9^ Permission to access these data was obtained from the police headquarters. By law, all road traffic crashes must be reported to the police within 24 hours. For the database, a road traffic crash was defined as a crash on a public highway or road that involved a vehicle and also involved personal injury or damage to property.^1^^,^^8^ Crashes were classified as involving one of four types of injury: (i) fatal (i.e. a victim died due to injuries sustained in the crash, irrespective of the time interval between the crash and death); (ii) serious (i.e. the crash resulted in one or more kinds of severe injury, such as bone fractures, damage to internal organs, severe burns, permanent impairment of vision or hearing or serious disfigurement); (iii) minor; or (iv) none (i.e. the crash did not cause any injury and resulted in only damage to vehicles).^9^ If more than one type of injury was present, the most serious type of injury was recorded.

Data were retrieved manually from the traffic police’s database and entered into an Excel spreadsheet (Microsoft, Redmond, United States of America) for analysis. Information on the size of the population of Sri Lanka in the middle of each year was obtained from the Department of Census and Statistics^13^ and used to calculate road traffic fatality and injury rates per 100 000 population. In addition, we obtained information on the total number of registered vehicles for the period 1938 to 2013 from the Department of Motor Traffic.^11^ We plotted the number of road traffic crashes, the population and the number of registered vehicles over the study period using Excel and R (R Foundation for Statistical Computing, Vienna, Austria). We analysed long-term trends in road traffic crashes, injuries and fatalities using the Joinpoint Regression Program Version 4.0.4 (National Cancer Institute, Bethesda, USA).^14^^,^^15^ Joinpoints are points in a time series at which statistically significant changes occur and joinpoint regression fits a series of joined straight lines between these joinpoints. The program starts with the simplest model of fit and tests several models, while increasing joinpoints, until a statistically significant fit is obtained. The program uses the Monte Carlo permutation method to test for significance.^14^^,^^15^ We reviewed the literature on Sri Lanka’s traffic and transportation history to identify events that might have contributed to the significant changes observed in the joinpoint analysis. Finally, to supplement our findings, we conducted an additional analysis for the period 1977 to 2013, during which it was possible to separate data on serious and minor injuries.

## Results

**Fig. 1:**
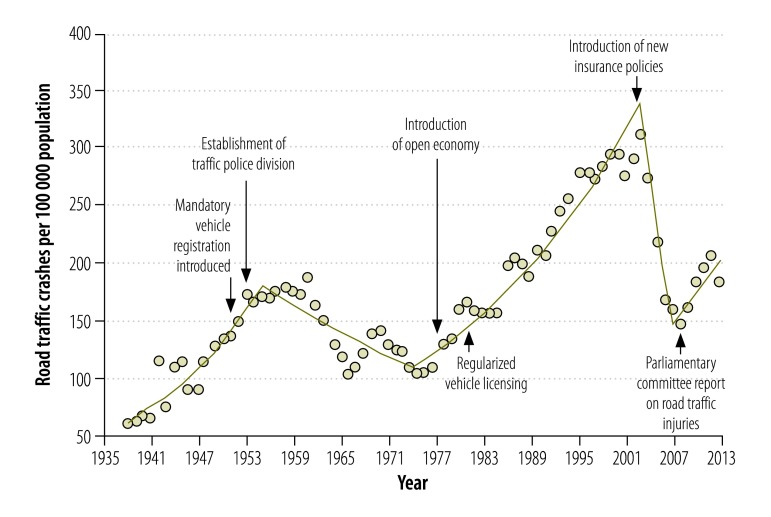
Road traffic crashes, Sri Lanka, 1938–2013

The incidence of road traffic crashes between 1938 and 2013 is shown in Fig. 1. There were substantial changes in the trend: for the best fitting model, there were four joinpoints, in 1955, 1974, 2003 and 2007, respectively (Table 1). Overall, road traffic crashes increased markedly from 61.2 per 100 000 population in 1938 to 183.6 per 100 000 in 2013 – a threefold increase. However, the increase was not continuous. Road traffic crashes increased steadily by 180% (from 61.2 to 170.8 per 100 000 population) between 1938 and 1955, but between 1955 and 1974, it decreased by 36% (from 170.8 to 109.1 per 100 000 population). Crashes increased again between 1974 and 2003, by 185% (from 109.1 to 310.7 per 100 000 population), but decreased between 2003 and 2007, by 49% (from 310.7 to 159.8 per 100000 population). Between 2007 and 2013, the annual percentage change was not significant. The highest incidence in the 75-year period was reported in 2003, at 310.7 per 100 000 population. 

**Table 1:** Model of best fit, joinpoint regression analysis of road traffic crashes, Sri Lanka, 1938–2013

Time segment	Lower endpoint, year	Upper endpoint, year	Annual percentage change (95% CI)
1	1938	1955	6.5 (5.3 to 7.6)
2	1955	1974	−2.5 (−3.5 to −1.5)
3	1974	2003	3.9 (3.4 to 4.4)
4	2003	2007	−18.6 (−30.4 to −4.8)
5	2007	2013	5.4 (0.0 to 11.2)

CI: confidence interval.

**Fig. 2:**
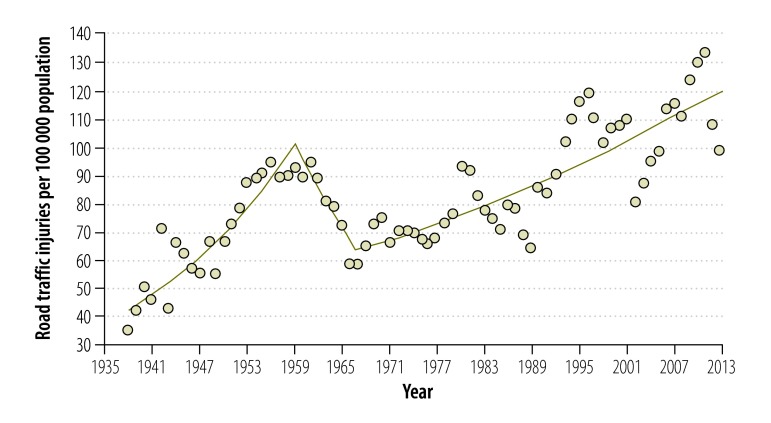
Road traffic injuries, Sri Lanka, 1938–2013

Fig. 2 shows the incidence of road traffic injuries between 1938 and 2013. Over the period, it increased from 35.1 to 98.6 per 100 000 population. The model of best fit had two joinpoints, in 1959 and 1967, respectively (Table 2). The road traffic injury rate increased until 1959 (to 92.5 per 100 000 population), decreased from 1959 to 1967 (to 58.1 per 100 000 population) and then increased again until 2013. However, during the latter half of the study period, annual rates deviated substantially from the trend line. 

**Table 2:** Model of best fit, joinpoint regression analysis of road traffic injuries, Sri Lanka, 1938–2013

Time segment	Lower endpoint, year	Upper endpoint, year	Annual percentage change (95% CI)
1	1938	1959	4.2 (3.3 to 5.1)
2	1959	1967	−5.6 (−9.9 to −1.1)
3	1967	2013	1.4 (1.1 to 1.7)

CI: confidence interval.

**Fig. 3:**
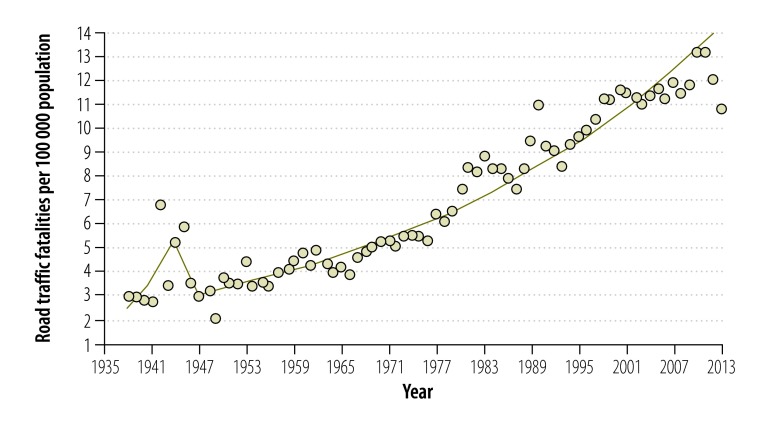
Road traffic fatalities, Sri Lanka, 1938–2013

Fig. 3 shows that road traffic fatalities increased markedly between 1938 and 2013, from 3.0 to 10.8 per 100 000 population. The joinpoint program indicated two join points, in 1944 (5.3 per 100 000 population) and 1947 (3.0 per 100 000 population), respectively (Table 3). Fatalities increased continuously from 1947 onwards.

**Table 3:** Model of best fit, joinpoint regression analysis of road traffic fatalities, Sri Lanka, 1938–2013

Time segment	Lower endpoint, year	Upper endpoint, year	Annual percentage change (95% CI)
1	1938	1944	13.2 (6.0 to 20.9)
2	1944	1947	−16.7 (−43.5 to 22.8)
3	1947	2013	2.3 (2.2 to 2.5)

CI: confidence interval.

**Fig. 4:**
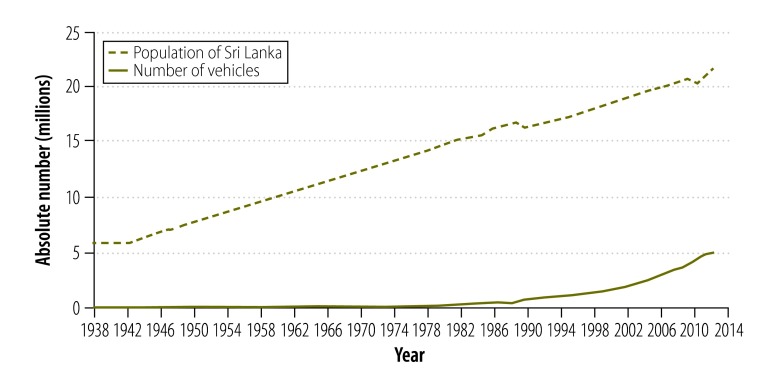
Population and absolute number of vehicles, Sri Lanka, 1938–2013

**Fig. 5:**
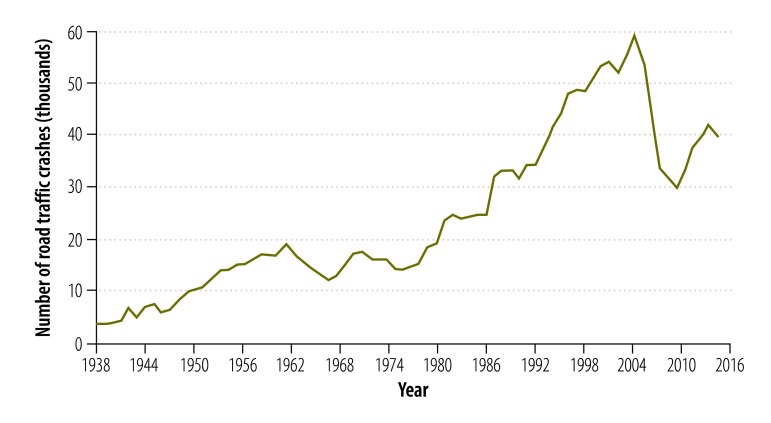
Absolute number of road traffic crashes, Sri Lanka, 1938–2013

**Fig. 6:**
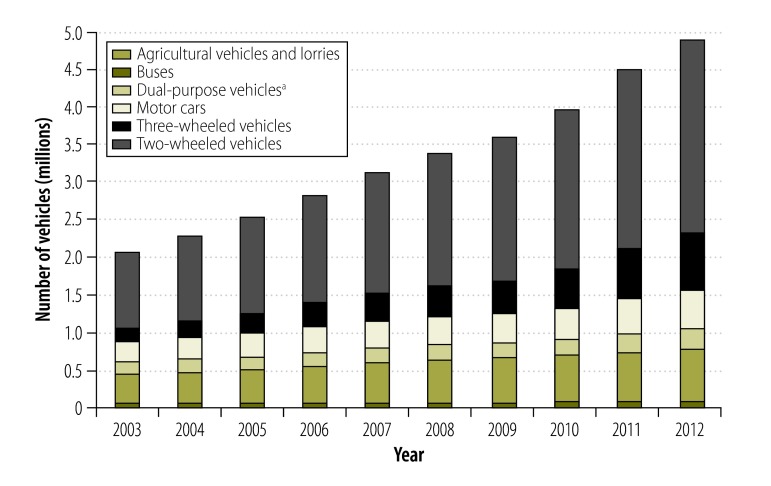
Registered vehicles, by type, Sri Lanka, 2003–2012

^a^ Dual-purpose vehicles carry both goods and people. Their gross vehicle weight does not exceed 3500 kg and their seating capacity is nine or less.

**Fig. 7:**
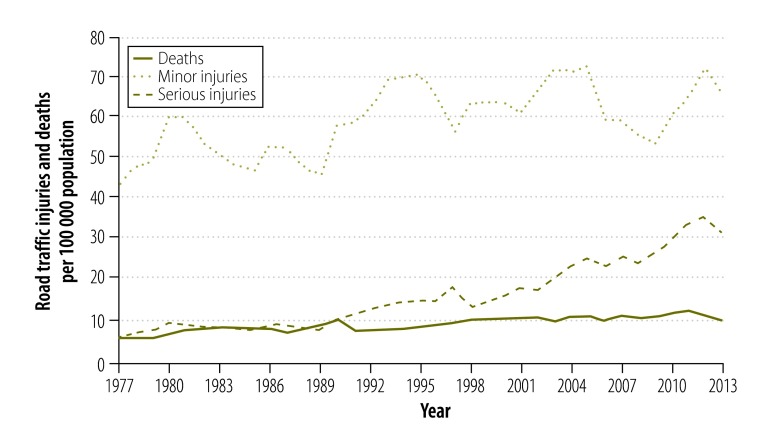
Deaths and injuries due to road traffic crashes, Sri Lanka, 1977–2013

Note: The graphs show deaths, serious injuries and minor injuries due to road traffic crashes reported to the police.

Fig. 4 and Fig. 5 show the absolute number of road traffic crashes and vehicles, and the population size over the study period. All three variables increased during the 75 years but the most marked increases in road traffic crashes and in the number of vehicles were observed in the second half of the study period. Fig. 6 shows the number of registered vehicles between 2003 and 2012, by vehicle type. Categorized data were not available before 2003. Fig. 7 shows the number of deaths, serious injuries and minor injuries due to road traffic crashes per 100 000 population that were reported to the police between 1977 and 2013. There was a marked increase in the number of serious injuries over the period, from 6.0 to 32.0 per 100 000. Although deaths increased steadily, the number of minor injuries fluctuated considerably.

## Discussion

Our analyses cover 75 years of road traffic crashes, injuries and fatalities in Sri Lanka. Although the global status report on road safety produced by the World Health Organization (WHO) in 2013 included details of road traffic fatalities in Sri Lanka between 2001 and 2010, it covered only a short period and did not contain a detailed discussion of the possible reasons for any increase.^16^ In addition, WHO’s 2004 world report on road traffic injury prevention discussed road traffic fatality trends in Asia between 1987 and 1995 but did not explain trends in individual countries.^1^

We found that road traffic crashes, injuries and fatalities in Sri Lanka all increased between 1938 and 2013. However, there were fluctuations over this time; and several factors are associated with these changes.

Road infrastructure developed rapidly between 1938 and 1955. During this period, the greater numbers of crashes may have been due to more frequent road use or to more vehicles on the roads.^1^^–^^3^ Also, during this period the country had a poorly regulated bus service and drivers competed for passengers and violated traffic laws – this behaviour may have increased the number of bus crashes and made roads less safe for other users. Moreover, although traffic laws were in place, law enforcement was likely to have been poor because Sri Lanka did not have a traffic police unit during this period. Monitoring traffic was one of the duties of general police units.^11^

Three factors might have contributed to the reduction in road traffic crashes between 1955 and 1974. First, a new traffic law was introduced in 1951 that required all motor vehicles to be registered with the Department of Motor Traffic. A vehicle’s roadworthiness had to be tested before registration.^12^ Second, in 1953, Sri Lanka established a traffic police division and increased the number of traffic police leading to better enforcement of traffic laws. This division also assisted the Inspector General of Police in introducing and implementing new traffic policies.^11^ Third, in 1958, the government nationalized the privately-owned bus service and improved the quality and availability of public transportation.^8^ Better and more accessible public transportation might have encouraged more people to travel by bus and discouraged the use of private vehicles.

The introduction of an open economic policy in Sri Lanka could have contributed to the increase in road traffic crashes observed after 1977. This policy made it easier to import vehicles and led to an exponential growth in their numbers:^17^ between 1977 and 2003 the number of vehicles increased by 2790%. The increase was greatest in motorized two- and three-wheeled vehicles,^11^ which were involved in more crashes than other vehicle types.^1^^–^^3^ During this period, there was no matching expansion of either the road infrastructure or the traffic police – this mismatch might have contributed to the marked increase in crashes observed between 1977 and 2003.

The reduction in road traffic crashes after 2003 might have been due to a smaller proportion of crashes being reported. Although Sri Lanka’s traffic law requires all road traffic crashes to be reported to the police, many drivers do not report those that involve only minor injuries or damage.^7^ In Sri Lanka, reporting crashes to the police can be exhausting: drivers have to spend many hours at the crash site or at a police station until the police complete the initial investigation. If a police report is required for insurance purposes, drivers may have to wait a few days.

To simplify procedures for drivers, Sri Lankan insurance companies introduced an on-site insurance payment policy in 2002. The so-called on-the-spot insurance scheme paid insurance claims without police reports.^18^ Although drivers benefited, the new policy probably led to substantial underreporting of road traffic crashes. Our data on road traffic injuries indicate that underreporting probably occurred: no reduction in road traffic injuries was observed between 2003 and 2007, when road traffic crashes appeared to be falling. People injured in road traffic crashes usually present to hospital emergency departments and hospitals report these injuries to the police based in the hospital, who in turn report them to the Police Statistics Unit.^19^ Therefore, although drivers might not have reported crashes to the police, associated injuries would have been reported through the hospitals.

It is important to note that the on-the-spot insurance scheme led not only to the underreporting of crashes but also enabled drivers involved in minor crashes to avoid police and legal procedures. These drivers could subsequently have been involved in further crashes, thereby making roads less safe for other drivers and pedestrians. To improve this situation, Sri Lanka should increase the traffic police workforce and require officers to reach accident sites quickly, to investigate and record all road traffic crashes and to provide on-site police reports for insurance purposes. Moreover, insurance companies should be required to obtain police reports before issuing insurance payments.

In 2007, a parliamentary committee was appointed to investigate reasons for the high number of road traffic injuries in Sri Lanka.^20^ There was an increase in road traffic crashes reported after 2007. The actions of this committee and the 2009 end of the civil war in the north-east may have both contributed to this increase in crashes reported. However, no amendments were made to the on-the-spot insurance scheme and the discrepancies between reported crashes and injuries continued. The parliamentary committee proposed several interventions to prevent road traffic crashes and a number have been implemented since 2010, including: computerizing driving licence registration; expanding the road network; displaying speed limits at the roadside; and training drivers. However, the available data do not allow us to test for any effects of these interventions.

The increasing trend in road traffic fatalities over the study period was much more consistent than those of road traffic crashes or injuries. This steady increase in road traffic fatalities has also been reported in other low- and middle-income countries, including Cambodia, India, Iraq, Myanmar, Nepal and Saudi Arabia.^16^^,^^21^ As in Sri Lanka, these increases have been associated with a rise in the number of vehicles, poor traffic law enforcement and underdeveloped road infrastructure.^1^^–^^3^^,^^21^^–^^23^

Our descriptive analysis of road traffic injuries between 1977 and 2013 showed that all types of injuries increased. However, there were greater fluctuations in minor injuries than in serious injuries or fatalities. The 1981 traffic laws that limited the number of passengers in motor vehicles and regularized the vehicle licencing process^10^^,^^11^ and better traffic law enforcement in 1995 may have influenced these trends. However, passenger limits, licencing and law enforcement do not seem to have had a long-term effect in reducing minor injuries. Underreporting due to the introduction of the on-spot insurance scheme could be a plausible cause for the fluctuations seen after 2003 and this hypothesis needs further research.

One limitation of our study was that we used traffic-police data, which included only road traffic crashes and injuries either recorded by the police or reported to them. However, although crashes were underreported after 2003, we were still able to draw important conclusions from the data available. Another limitation was the paucity of scientific studies of the reasons underlying the fluctuations in road traffic crashes in Sri Lanka. A strength of our study was the use of joinpoint regression to analyse long-term trends in road traffic crashes, injuries and fatalities as has been done in high-income countries.^14^^,^^15^^,^^24^^,^^25^

Sri Lanka’s burden of road traffic injuries could be reduced by better enforcement of traffic laws, restrictions on the importation of two- and three-wheeled motor vehicles and the introduction of new policies to improve road safety.
